# MAVS-MKK7-JNK2 Defines a Novel Apoptotic Signaling Pathway during Viral Infection

**DOI:** 10.1371/journal.ppat.1004020

**Published:** 2014-03-20

**Authors:** Yuefeng Huang, Heng Liu, Senlin Li, Yijun Tang, Bo Wei, Huansha Yu, Chen Wang

**Affiliations:** State Key Laboratory of Cell Biology, Institute of Biochemistry and Cell Biology, Shanghai Institutes for Biological Sciences, Chinese Academy of Sciences, Shanghai, China; Kantonal Hospital St. Gallen, Switzerland

## Abstract

Viral infection induces innate immunity and apoptosis. Apoptosis is an effective means to sacrifice virus-infected host cells and therefore restrict the spread of pathogens. However, the underlying mechanisms of this process are still poorly understood. Here, we show that the mitochondrial antiviral signaling protein (MAVS/VISA/Cardif/IPS-1) is critical for SeV (Sendai virus)-induced apoptosis. MAVS specifically activates c-Jun N-terminal kinase 2 (JNK2) but not other MAP kinases. Jnk2−/− cells, but not Jnk1−/− cells, are unable to initiate virus-induced apoptosis and SeV further fails to trigger apoptosis in MAPK kinase 7 (MKK7) knockout (Mkk7−/−) cells. Mechanistically, MAVS recruits MKK7 onto mitochondria via its 3D domain, which subsequently phosphorylates JNK2 and thus activates the apoptosis pathway. Consistently, Jnk2−/− mice, but not Jnk1−/− mice, display marked inflammatory injury in lung and liver after viral challenge. Collectively, we have identified a novel signaling pathway, involving MAVS-MKK7-JNK2, which mediates virus-induced apoptosis and highlights the indispensable role of mitochondrial outer membrane in host defenses.

## Author Summary

The mitochondrial antiviral signaling protein (MAVS/VISA/Cardif/IPS-1) is critical for the innate immune response during viral infection, and its function has been well documented in mediating type I interferon production. In this study, we revealed the essential role of MAVS in virus-induced apoptosis, independent of Retinoic acid-Inducible Gene I (RIG-I) signaling. Upon viral infection, MAVS recruits MKK7 onto mitochondria, followed by MKK7 induced activation of JNK2, which subsequently initiates apoptosis. Importantly, we have clearly differentiated the roles of JNK2 versus JNK1, and MKK7 versus MKK4 in virus-induced apoptosis. Thus, we define a novel apoptotic signaling pathway, involving MAVS-MKK7-JNK2, which sheds a new perspective on the crosstalk between the antiviral and apoptotic signaling pathways in innate immunity.

## Introduction

The induction of innate immunity upon viral infection represents the first line of host defense against microbe invasion. During infection with a RNA virus, the mitochondrial antiviral signaling protein (MAVS/VISA/Cardif/IPS-1) has been recently uncovered to seed a critical protein complex on the mitochondrial outer membrane [1]–[4]. This signalosome consists of TNFR-associated factors (TRAF2/3/6) [5], TNFR-associated death domain protein (TRADD) [6], translocase of outer mitochondrial membrane 70 (TOM70) [7], ubiquitously expressed transcript (UXT-V1) [8], Autophagy proteins (Atg5/Atg12) [9], Mitofusin-2 (Mfn2) [10] *et.al*. The MAVS signalosome activates TANK-binding kinase 1 (TBK1), which then phosphorylates interferon (IFN) regulatory factor 3(IRF3). In addition, the MAVS signalosome can also activate the IκB kinase (IKK) complex and nuclear factor κB (NF-κB). Synergistically, these transcription factors (IRF3 and NF-κB) induce the early production of type I interferons and inflammatory cytokines [11].

The induction of apoptosis following viral infection is another effective means by which the host attempts to restrict the spread of pathogens by sacrificing the virus-infected cells [12]. Several recent studies suggested that MAVS mediates virus-induced cell apoptosis and that this function is independent of the interferon pathway. MAVS, as well as UXT-V1/V2, have been reported to promote the clearance of infected cells [13]. MAVS overexpression causes cell apoptosis and several viral proteins, such as hepatitis C virus NS3/4A and the Severe Acute Respiratory Syndrome coronavirus (SARS-CoV) nonstructural protein (NSP15), are inhibitors of the MAVS-induced apoptosis [14]. Borna disease virus (BDV) X protein can also interact with MAVS to inhibit MAVS-mediated apoptosis [15]. In addition, MAVS decreases the K48-linked ubiquitination of voltage-dependent anion channel 1(VDAV1) [16]. Activation of MAVS by Bunyavirus infection upregulates the adaptor protein SARM1, leading to neuronal death [17]. However, the molecular mechanism of how MAVS regulates apoptosis remains poorly understood.

It is intriguing to understand the roles of mitochondria in virus-induced apoptosis, in particular, elucidation of specific apoptotic signaling pathways. Sendai virus (SeV), Newcastle disease virus (NDV) and Vesicular stomatitis virus (VSV) are enveloped negative-strand RNA viruses, which are able to trigger acute infection in rodents and induce robust cell apoptosis [18]. As such, these infections represent effective models for studying virus-induced apoptosis. Due to the central role of mitochondria in apoptosis and the mitochondrial localization of MAVS, it is important to explore the putative function of MAVS in the apoptotic signaling pathway. This work will be instrumental towards understanding the molecular mechanisms regarding host decisions on cell survival and apoptosis, and shedding further light on the cross-talk between innate immunity and apoptosis.

In this study, we report that MAVS plays an essential role in virus-induced apoptosis. Upon SeV infection, MAVS recruits MAPK kinase 7 (MKK7) onto mitochondria via its 3D domain, which specifically activates c-Jun N-terminal kinase 2 (JNK2) and triggers cell apoptosis. SeV was unable to trigger apoptosis in either *Mavs* ^−/−^ cells or *Mkk7* ^−/−^ cells. In addition, *Jnk2* ^−/−^ cells, but not *Jnk1* ^−/−^ cells, failed to initiate the virus-induced apoptosis. Interestingly, RIG-I and MDA5 (melanoma differentiation-associated protein 5) were not required for this apoptosis. Consistently, *Jnk2* ^−/−^ mice, but not *Jnk1* ^−/−^ mice, displayed marked inflammatory injury in both lung and liver after viral challenge. This study identifies a novel signaling pathway, MAVS-MKK7-JNK2, to mediate virus-induced apoptosis, revealing the indispensable role of the mitochondrial outer membrane in host defense.

## Results

### MAVS induces JNK activation upon viral infection

**Figure 1:**
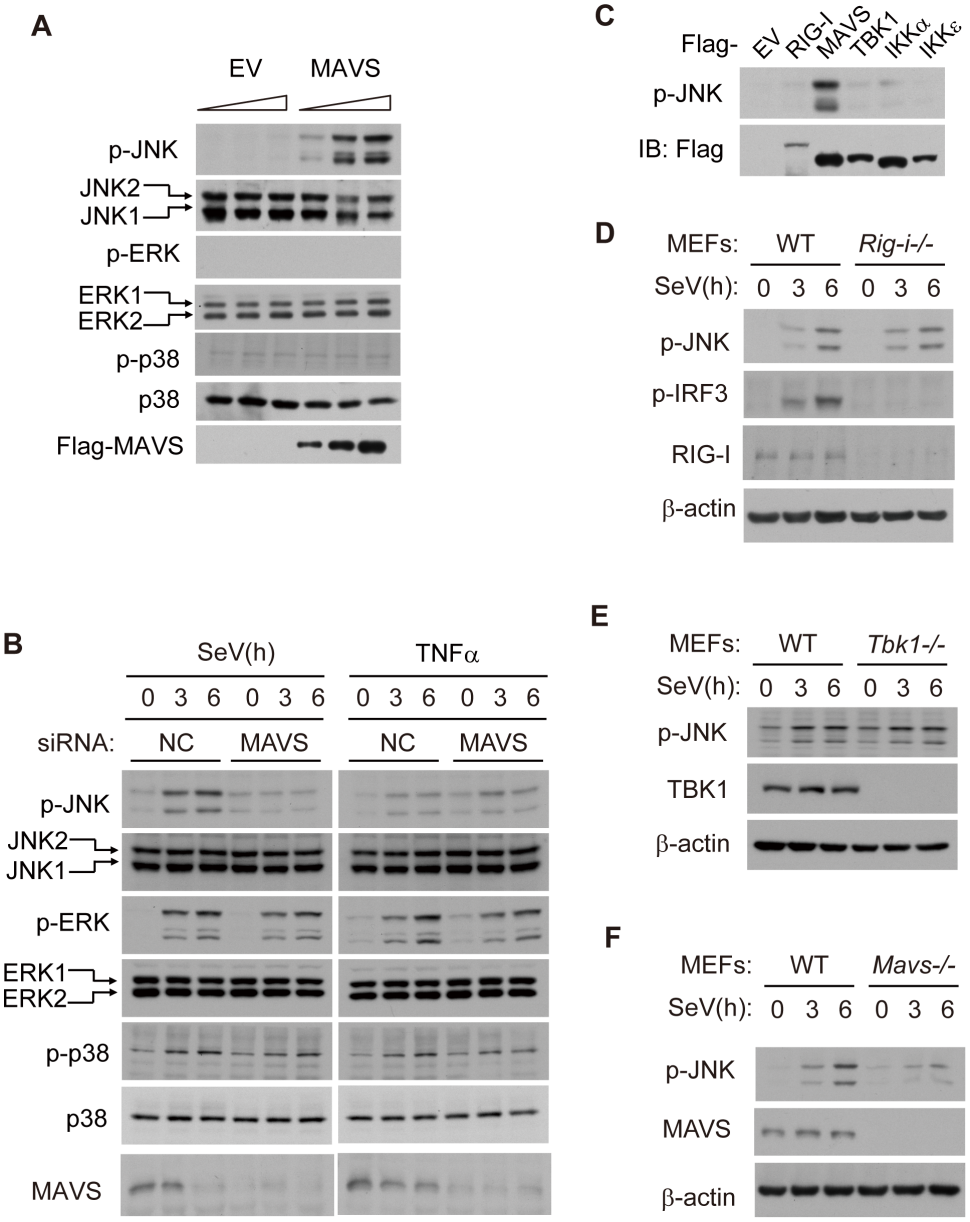
MAVS induces JNK activation during viral infection. (***A***) HEK293 cells were transfected with the Flag-MAVS plasmid or empty pCDNA3.0 vector (EV) at dose gradient (0, 1 or 2 µg/well in a 12-well plate) for 24 hours, cell lysates were collected for western blot analysis using the indicated antibodies. (***B***) HEK293 cells were transfected with MAVS siRNA or negative control siRNA and then treated with SeV (MOI = 1) or TNF-α (10 ng/ml) for the indicated times. Whole cell lysates were collected for western blot analysis of p-JNK, JNK, p-ERK, ERK, p-p38, p38 and MAVS. NC, negative control, MOI, multiplicity of infection, p-, phosphorylated. (***C***) HEK293 cells were transfected with EV or with plasmids of the indicated antiviral signaling components. Whole cell lysates were collected for western blot analysis. Anti-p-JNK antibody was used to measure the level of JNK phosphorylation, and anti-Flag antibody was used to determine the transfecting efficiency. (***D, E*** *and* ***F***) Wild type (WT), RIG-I knockout (*Rig-i^−/−^*), TBK1 knockout (*Tbk1^−/−^*) or MAVS knockout (*Mavs^−/−^*) MEF cells were treated with SeV (MOI = 4) for the indicated times. Cell lysates were immunoblotted for phosphorylated JNK and corresponding deficient protein.

Given that MAVS robustly activates TBK1 and IKK kinases, we wondered if MAVS could influence Mitogen-Activated Protein Kinase (MAPK) signaling. To explore this possibility, we overexpressed MAVS in HEK293 cells and then checked the activation of JNK, extracelluar signal-regulated kinase (ERK) and p38. Interestingly, the phosphorylation of JNK exhibited a dose-dependence on the expression of MAVS, whereas similar effects did not apply to either ERK or p38 (Figure 1A), suggesting that MAVS may specifically activate JNK. Using siRNA to knock down endogenous MAVS, we further explored the SeV-induced activation of JNK. As expected, SeV could induce the phosphorylation of all the MAP kinases (JNK, ERK and p38). Notably, knockdown of MAVS appeared to attenuate JNK activation, whereas this did not influence the activation of ERK or p38 (Figure 1B). In addition, the knock down of MAVS did not affect tumor necrosis factor alpha (TNFα)-triggered phosphorylation of JNK, ERK or p38 (Figure 1B). These observations suggest that virus-induced activation of JNK is dependent on MAVS.

Considering the critical function of MAVS in innate immunity, we tested if the relevant signaling proteins could also activate JNK. Ectopic-expression of RIG-I, TBK1, IKKα or IKKε did not influence JNK phosphorylation (Figure 1C). RIG-I and MDA5 are the viral nucleic acid sensors and are both upstream of MAVS signaling. We used *Rig-i^−/−^* MEF cells to determine whether RIG-I also mediated JNK phosphorylation. Surprisingly, knockout of RIG-I didn't influence the SeV-triggered JNK phosphorylation, although it did abrogate IRF3 activation (Figure 1D). We also prepared siRNA sets to specifically knock down RIG-I or MDA5. The results showed that a decrease in either RIG-I or MDA5 did not impact SeV-induced JNK phosphorylation (Figure S1A). The absence of TBK1 also had no effect on JNK phosphorylation (Figure 1E). In contrast, MAVS deficiency completely blocked SeV-induced JNK activation (Figure 1F). Taken together, the SeV-induced activation of JNK is dependent on MAVS, yet independent of RIG-I/MDA5 and TBK1/IKK. These results suggest that MAVS is the converging point for activating JNK, TBK1 and IKK during viral infection.

### JNK2, but not JNK1, is essential for virus-induced cell apoptosis

**Figure 2:**
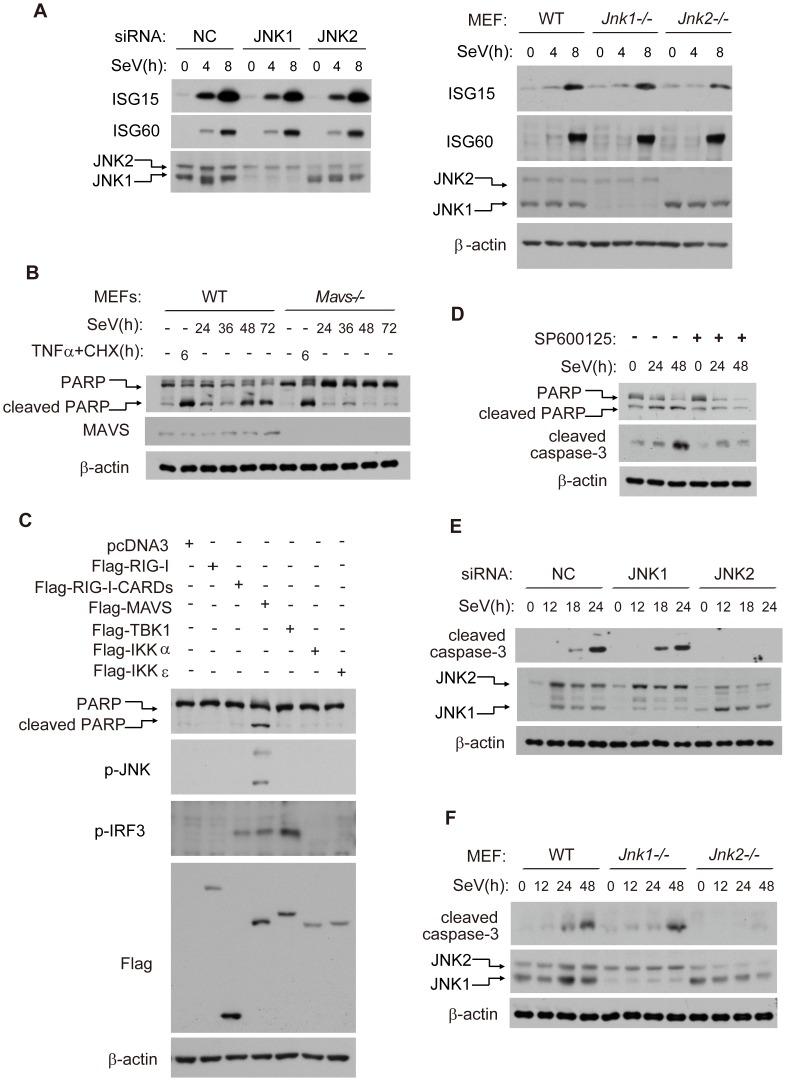
JNK2, but not JNK1, is essential for virus-induced apoptosis. (***A***) Control, JNK1 and JNK2 siRNA knock-down HEK293 cells (***left***), or wild type, *Jnk1* ^−/−^ and *Jnk2* ^−/−^ MEF cells (***right***), were treated with SeV (for HEK293 MOI = 1, for MEF MOI = 4) for the indicated times. Cell lysates were analyzed by western blot, probing for ISG15, ISG60, JNK1 and JNK2 with the indicated antibodies. (***B***) Wild type and *Mavs^−/−^* MEF cells were treated with SeV (MOI = 4), or TNF-α (10 ng/ml) plus cycloheximide (CHX, 10 µg/ml) for the indicated times. Cell lysates were collected for western blot analysis using anti-PARP antibody to determine cell apoptosis and using anti-MAVS antibody to measure the deficiency of MAVS protein. (***C***) HEK293 cells were transfected with the indicated plasmids and 24 hours later, cell lysates were collected for western blot analysis of PARP, phosphorylated JNK, phosphorylated IRF3, Flag-tagged proteins and β-actin. (***D***) HEK293 cells were treated by SeV (MOI = 1) with or without JNK kinase inhibitor SP600125 (5 µM). Cell lysates were collected for western blot analysis of PARP, cleaved caspase-3 and β-actin to probe for cell apoptosis. (***E***) Control, JNK1 or JNK2 knocked down HEK293 cells were treated with SeV (MOI = 1) for the indicated times. Cell lysates were collected for western blot analysis to measure cell apoptosis using the indicated antibodies. (***F***) Wild type, *Jnk1^−/−^* or *Jnk2^−/−^* MEF cells were treated with SeV (MOI = 4) for the indicated times. Cell lysates were collected for western blot analysis.

We went on to explore whether JNK could modulate type I interferon signaling. Interestingly, we observed no difference of SeV-induced Interferon Stimulated Gene 15/60(ISG15/ISG60) production amongst control, JNK1 deficiency or JNK2 deficiency, using either siRNA knock down in HEK293 cells (Figure 2A, left) or in knockout mouse embryonic fibroblast cells (MEFs) (Figure 2A, right), indicating that JNK1/2 are dispensable for virus-induced interferon β (IFN-β) signaling.

In order to test whether MAVS plays a role in virus-induced apoptosis, we measured cell apoptosis by monitoring the apoptosis marker poly ADP ribose polymerase (PARP) in *Mavs* ^+/+^ and *Mavs* ^−/−^ MEFs. Notably, *Mavs* ^−/−^ cell had much less apoptosis than *Mavs* ^+/+^ cells following SeV challenge. In contrast, TNFα/CHX (cycloheximide) induced comparable level of apoptosis in *Mavs* ^+/+^ and *Mavs* ^−/−^ cells (Figure 2B). These results indicate that MAVS specifically modulates virus-induced apoptosis. In addition, ectopic-expression of MAVS potentiated JNK phosphorylation and apoptosis, whereas RIG-I (full length), RIG-I-CARDs (constitutively activated form of RIG-I), TBK1, IKKα and IKKε failed to do so (Figure 2C). We also measured SeV-induced apoptosis in *Rig-i^−/−^* MEFs. Consistently, there was no difference in the cleavage of PARP or caspase-3, between RIG-I knockout and wild type control (Figure S1B).

Based on these results, we hypothesized that the MAVS-dependent activation of JNK was linked to virus-induced apoptosis. It was observed that the general inhibitor for JNK1/2(SP600125) markedly attenuated the SeV-induced PARP/caspase-3 cleavages (Figure 2D). Consistently, the caspase inhibitor Z-VAD effectively blocked the PARP/caspase-3 cleavages, whereas the inhibitor did not affect the phosphorylation of JNKs upon SeV stimulation (Figure S2A and S2B), suggesting that JNK activation is primary, not secondary to cell apoptosis. Unexpectedly, knock down of endogenous JNK2 alone significantly attenuated the SeV-induced PARP/caspase-3 cleavages, whereas knockdown of JNK1 alone did not appear to influence apoptosis (Figure 2E). These observations were further substantiated by using *Jnk1* ^−/−^ and *Jnk2* ^−/−^ MEF cells after SeV infection (Figure 2F) or VSV infection (Figure S2C). Collectively, these data differentiate the functions of JNK1 and JNK2, revealing the specific role of JNK2 in SeV-induced apoptosis.

Since both TRADD and TRAF proteins are known components of the mitochondrial MAVS antiviral signalosome, we probed the role of TRADD and TRAFs in virus-induced JNK activation and subsequent apoptosis. Interestingly, knocking down TRADD or TRAFs had no observable influence on either JNK activation or apoptosis upon SeV infection (Figure S3), suggesting that TRADD and TRAFs are not involved in virus-induced MAVS/MKK7/JNK2 signaling.

### MKK7 functionally links MAVS to JNK2 in virus-induced apoptosis

**Figure 3:**
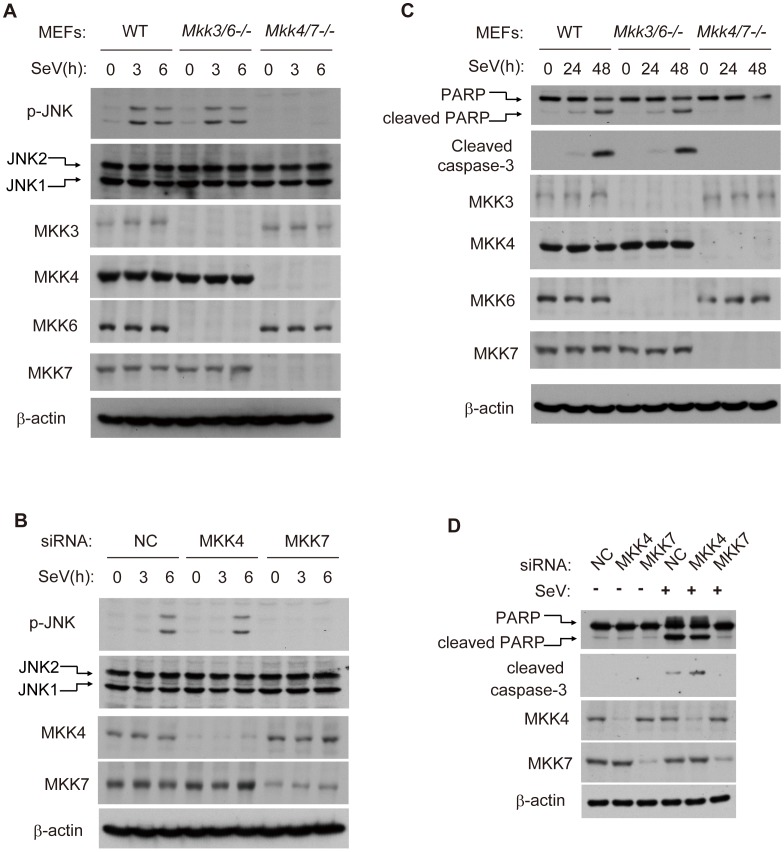
MKK7 functionally links MAVS to JNK2 during virus-induced apoptosis. (***A***) Wild type, *Mkk3/6* ^−/−^ or *Mkk4/7* ^−/−^ MEF cells were treated with SeV (MOI = 4) for the indicated times. Cell lysates were collected for western blot analysis using anti-JNK, anti-p-JNK, anti-MKK3, anti-MKK4, anti-MKK6, anti-MKK7 and anti-β-actin antibodies. (***B***) HEK293 cells were transfected with NC, MKK4 and MKK7 siRNAs respectively for 48 hours and then treated by SeV (MOI = 1) for the indicated times. Cell lysates were collected for western blot analysis of p-JNK, JNK, MKK4, MKK7 and β-actin. (***C***) Wild type, *Mkk3/6* ^−/−^ or *Mkk4/7* ^−/−^ MEF cells were treated with SeV (MOI = 4) for the indicated times. Cell apoptosis was determined by western blot analysis of activated PARP and cleaved caspase-3. (***D***) HEK293 cells were transfected with NC, MKK4 and MKK7 siRNAs for 48 hours and then treated with SeV (MOI = 1) for 24 hours. Cell lysates were collected for western blot analysis of PARP and cleaved caspase-3 to measure cell apoptosis. *Mkk3/6* ^−/−^, *Mkk3* and *Mkk6* double knockout; *Mkk4/7* ^−/−^, *Mkk4* and *Mkk7* double knockout.

MKK4 (MAPK kinase 4) and MKK7 are potential upstream kinases for JNK1 and JNK2 [19]. Given that both MKK4 and MKK7 could phosphorylate JNK1/2 in response to TNF-α, growth factors, DNA damage *et.al*, we tested whether MKK4/7 is essential for the SeV-induced JNK phosphorylation. To do this, we used *Mkk4/7* ^−/−^ or *Mkk3/6* ^−/−^ double knockout MEF cells. As expected, JNK1/2 phosphorylation was abolished in *Mkk4/7* ^−/−^ cells, whereas this was not the case in *Mkk3/6* ^−/−^ cells (Figure 3A). In addition, we individually knocked down MKK4 and MKK7 in HEK293 cells. MKK7, but not MKK4, was both necessary and sufficient for JNK phosphorylation (Figure 3B). Consistent with these results, SeV-induced apoptosis was significantly impaired in *Mkk4/7* ^−/−^ MEF cells, in contrast to *Mkk3/6* ^−/−^ MEF and wild type cells (Figure 3C). The specific knock down of MKK7, but not MMK4, further attenuated apoptosis in HEK293 cells (Figure 3D). Collectively, our results reveal MKK7 as the essential signal transducer for SeV-induced apoptosis.

### Viral infection triggers MKK7 to bind MAVS on mitochondria

**Figure 4:**
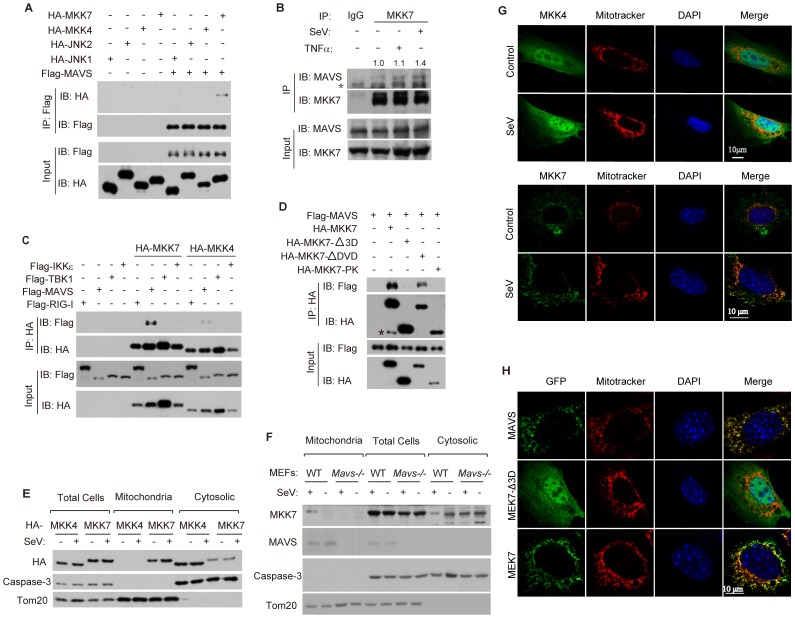
Viral infection triggers MKK7 to bind MAVS on mitochondria. (***A***) HEK293T cells were transfected with the indicated combination of plasmids. Cell lysates were immunoprecipitated using anti-Flag antibody. The immunoprecipitated proteins and input were detected by western blot analysis using anti-HA and anti-Flag antibodies. IP, immunoprecipitation. (***B***) HEK293 cells were treated with SeV (MOI = 1) or TNF-α (10 ng/ml) for 6 hours. Cell lysates were immunoprecipitated using anti-MKK7 antibody or normal rabbit IgG, followed by western blot analysis with anti-MAVS and anti-MKK7 antibodies. The immunoprecipitated MAVS was quantified relatively and the numeric values are shown on the top of the MAVS blot. Asterisk indicates a cross reaction band. (***C***) HEK293T cells were transfected with the indicated combinations of plasmids for 24 hours. Cell lysates were immunoprecipitated with anti-HA antibody, followed by western blot analysis with anti-HA and anti-Flag antibodies. (***D***) HA-tagged MKK7 or its truncation mutants were transfected into HEK293T cells along with Flag-MAVS for 24 hours. Cell lysates were immunoprecipitated with anti-HA antibody, followed by western blot analysis with anti-HA and anti-Flag antibodies. Asterisk indicates a cross reaction band. (***E***) HEK293 cells were transfected with HA-MKK4 or HA-MKK7 plasmids for 24 hours and then stimulated with or without SeV (MOI = 1) for 6 hours. Total cell lysates as well as mitochondrial and cytosolic fractions (obtained using subcellular fractionation) were probed with anti-HA, anti-caspase-3 (full length) and anti-Tom20 antibodies. Full length caspase-3 and Tom20 were applied to indicate the accuracy of fractionation. (F) Wild type or *Mavs^−/−^* MEF cells were stimulated with or without SeV (MOI = 4) for 6 hours. Subcellular fractionation was performed as described in ***E*** and the fractions were probed with anti-MKK7, anti-MAVS, anti-caspase-3(full length), and anti-Tom20 antibodies. (***G***) MEF cells were treated with or without SeV (MOI = 4) for 6 hours, The cells were then stained with the indicated antibodies and imaged by confocal microscopy. (***H***) MEF cells were transfected with either EGFP-MAVS, EGFP-MKK7-Δ3D, or EGFP-MKK7 and then treated with SeV (MOI = 4) for 6 hours. Cells were stained with Mitotracker and DAPI, and then imaged by confocal microscopy.

To elucidate the mechanism of MAVS-dependent activation of JNK2, we tested the potential interactions between MAVS and JNK1, JNK2, MKK4, MKK7, respectively. It was found that only MKK7 could interact with MAVS, whereas JNK1, JNK2 or MKK4 failed to do so (Figure 4A). We also confirmed the endogenous interaction between MAVS and MKK7. Notably, this endogenous interaction was markedly enhanced upon SeV infection (Figure 4B). In addition, MKK7 could not bind RIG-I, TBK1 or IKKε (Figure 4C). MKK7 was also unable to bind to MAVS-ΔTM, which is deprived of the trans-membrane domain(TM) and is localized inside the cytoplasm (Figure S4), suggesting that the trans-membrane domain of MAVS is important for its interaction with MKK7.

To further investigate the MAVS-MKK7 interaction, we generated three truncated mutants of MKK7, which were MKK7-Δ3D (lack of 3D domain), MKK7-ΔDVD (lack of DVD domain) and MKK7-PK (protein kinase domain only; lack of 3D, DVD and C-terminal) (Figure S5). We mapped the interaction between MAVS and the mutants. Only MKK7-ΔDVD, neither MKK7-Δ3D nor MKK7-PK could bind MAVS (Figure 4D), indicating that 3D domain mediates the MKK7-MAVS interaction.

Since MAVS is anchored on the mitochondrial outer membrane, we reasoned that MKK7 could dynamically be recruited onto mitochondria upon virus infection. Subcellular fractionation and confocal immunofluorescence revealed that a sub-pool of MKK7 constitutively co-localized with mitochondria, whereas no co-localization was evident with MKK4 (Figure 4E and 4G). Importantly, the presence of MKK7 on mitochondria apparently increased upon SeV stimulation, while MKK4 could not do so (Figure 4E and 4G), suggesting that MKK7 dynamically translocates onto the mitochondria upon virus infection. In *Mavs^−/−^* cells, MKK7 lost the ability to localize to mitochondria (Figure 4F), indicating this translocation is MAVS-dependent. In addition, MKK7-Δ3D, which lacks the 3D domain and is unable to bind MAVS, could not translocate onto mitochondria (Figure 4H), suggesting that the recruitment of MKK7 onto mitochondria depends on its interaction with MAVS.

### MAVS-MKK7-JNK2 defines a novel apoptotic signaling pathway

**Figure 5:**
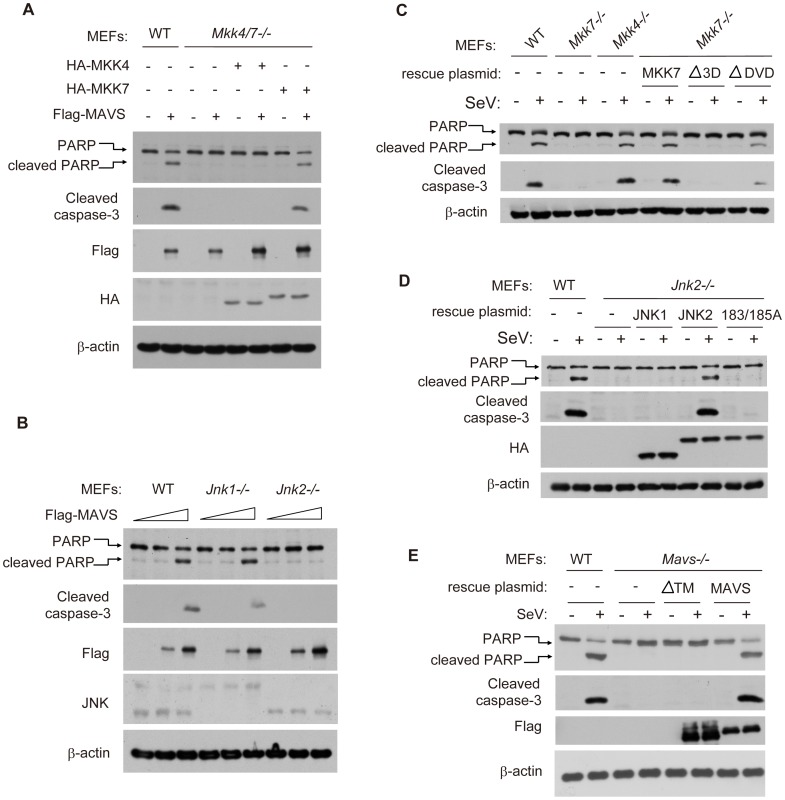
MAVS-MKK7-JNK2 represents a novel apoptotic signaling cascade. (***A***) Wild type and *Mkk4/7* ^−/−^ MEF cells were transfected with the indicated combinations of plasmids for 24 hours. *Mkk4/7* ^−/−^ MEFs with HA-MKK4 ectopic-expression were used to mimic *Mkk7* ^−/−^ and *Mkk4/7* ^−/−^ MEFs with HA-MKK7 ectopic-expression were used to mimic *Mkk4* ^−/−^. Cell lysates were collected for western blot analysis using the indicated antibodies. (***B***) Wild type, *Jnk1* ^−/−^ or *Jnk2* ^−/−^ MEF cells were transfected with the plasmid Flag-MAVS at a dose gradient (0, 2 and 4 µg/well in a 12-well plate). Cell lysates were collected for western blot analysis using the indicated antibodies. (***C***) Wild type or truncation mutants of MKK7 were re-introduced into “*Mkk7* ^−/−^ MEF cells” for 24 hours and the cells were then infected with SeV for 48 hours. Cell apoptosis was measured by western blot analysis of PARP and cleaved caspase-3. (***D***) HA-tagged JNK1, JNK2 and mutated JNK2(183/185A) were re-introduced into *Jnk2* ^−/−^ MEF cells, followed by similar analysis as described in ***C***. (***E***) Flag-tagged wild type MAVS and truncation mutant MAVS-ΔTM were re-introduced into *Mavs* ^−/−^ MEF cells, followed by similar analysis as described in ***C***.

To delineate the topology of apoptosis signaling, we re-introduced MKK4 or MKK7 into the *Mkk4/7* ^−/−^ double knockout MEFs and then monitored for apoptosis in these cells. It was observed that MKK7, but not MKK4, could rescue the MAVS-induced PARP and caspase-3 cleavages (Figure 5A), indicating that MKK7 functions downstream of MAVS. In addition, MAVS could induce the same apoptosis in *Jnk1* ^−/−^ MEF cells, but not in *Jnk2* ^−/−^ MEF cells (Figure 5B).

Furthermore, we re-introduced MKK4 or MKK7 respectively into the *Mkk4/7* ^−/−^ double knockout MEF cells, and accordingly named them as *Mkk*7^−/−^ or *Mkk4* ^−/−^ cells. Consistently, ectopic-expression of wild type MKK7 could rescue the SeV-induced apoptosis in *Mkk*7^−/−^ cells. However, MKK7-Δ3D could not (Figure 5C), which is consistent with the inability of MKK7-Δ3D to bind MAVS. MKK7-ΔDVD could partially rescue cell apoptosis due to its interaction with MAVS (Figure 4D and Figure 5C), implicating the functional interaction of MAVS and MKK7.

Biochemically, MKK7 phosphorylates JNK2 at 183-threonine (T) and 185-tyrosine (Y). We generated JNK2(183/185A) with both 183-threonine (T) and 185-tyrosine(Y) mutated to alanine and found that JNK2(183/185A) could not rescue the apoptosis in *Jnk2* ^−/−^ MEF cells, in comparison to JNK2(WT) (Figure 5D). Likewise, MAVS-ΔTM could not rescue the SeV-induced apoptosis in *Mavs* ^−/−^ MEF cells, indicating that it is necessary to form the signaling complex on mitochondria (Figure 5E). Taken together, MAVS→MKK7→JNK2 defines a novel apoptotic signaling pathway during viral infection.

### JNK2 protects mice against viral invasion

**Figure 6:**
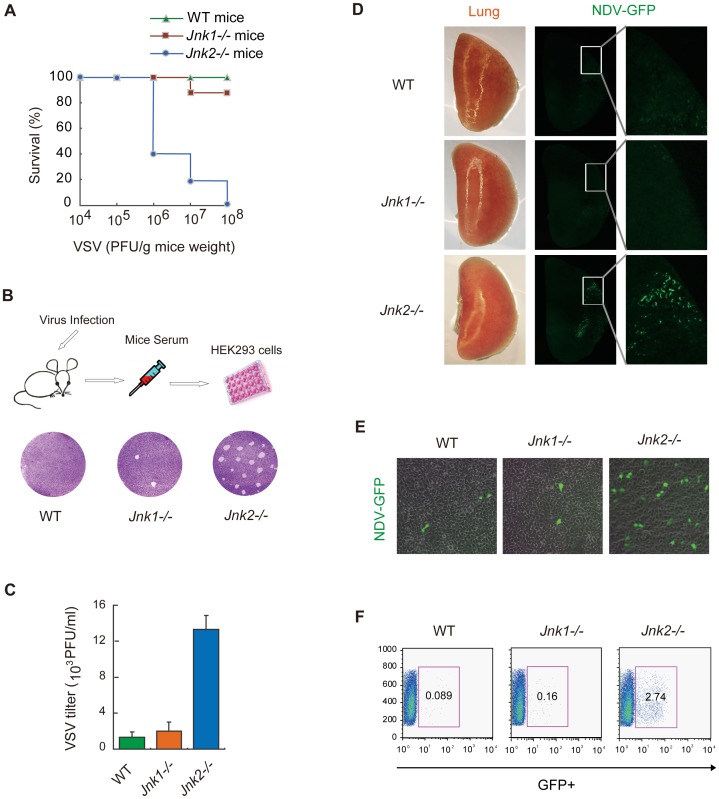
JNK2, but not JNK1, protects mice against viral infection *in vivo*. (***A***) Wild type C57BL/6, *Jnk1* ^−/−^ or *Jnk2* ^−/−^ mice (12 mice in each group) were infected with VSV at the indicated doses via nasal dripping (liquid volume≤20 µL). Survival rates of the mice were monitored at 5 days after infection. (***B***) The mice were intranasally challenged with VSV (10^6^ PFU/g mouse weight). Two days after infection, serum was collected from the mice. Equal volumes (10 µL) of serum were added to HEK293 cells culture media and incubated for 24 hours, followed by plaque assay using crystal violet staining. Viral plaques were observed by microscopy and the viral titer was calculated. (***C***) Data from B presented as means±SD (n = 3). (***D***) Wild type C57BL/6, *Jnk1* ^−/−^ or *Jnk2* ^−/−^ mice were infected with NDV-GFP (10^6^ PFU/g mouse weight). Two days after viral challenge, lungs were collected for fluorescence stereomicroscopy to observe NDV-GFP *in situ*. (***E***) Serum was collected at 2 days after NDV-GFP infection and equal volumes (100 µL) of serum were added to HEK293 cells culture media. Eighteen hours later, the NDV-GFP infected cells were observed by fluorescence microscopy. (***F***) GFP-positive cells from ***E*** were quantified by flow cytometry.

To determine the *in vivo* function of JNK2, we employed the vesicular stomatitis virus (VSV) infection model using wild type, *Jnk1* ^−/−^ and *Jnk2* ^−/−^ mice. The mortality of the *Jnk2* ^−/−^ mice was proportional to the amount of VSV administrated. Almost all of the *Jnk2* ^−/−^ mice died when high dose of VSV was used (Figure 6A). In contrast, only a small fraction of the *Jnk1* ^−/−^ mice died and no wild type mice died, under the same conditions (Figure 6A). Two days after VSV infection, we prepared serum from the mice and performed plaque assays in HEK293 cells (Figure 6B, upper). Consistently, VSV replicated much more robustly in *Jnk2* ^−/−^ mice (Figure 6B, lower). Quantification revealed that the viral titer of VSV in *Jnk2* ^−/−^ mice was 7–10 fold higher than that in *Jnk1* ^−/−^ or wild-type mice (Figure 6C). These results indicate that JNK2 could protect mice against the VSV infection, whereas JNK1 was dispensable.

As a second viral infection model to investigate the role of JNK2, GFP-labeled Newcastle Disease Virus (NDV-GFP) was used to challenge the mice intranasally. Two days after infection, the lungs of the wild-type, *Jnk1* ^−/−^ and *Jnk2* ^−/−^ mice were respectively imaged *in situ* by fluorescence microscope. Strikingly, NDV-GFP was markedly observed in the lung of the *Jnk2* ^−/−^ mice, whereas no or only marginal NDV-GFP were detected in the lungs of wild-type or *Jnk1* ^−/−^ mice (Figure 6D). Serum was also collected from the mice and the NDV-GFP titers were monitored by infecting HEK293 cells and using fluorescence microscopy (Figure 6E) and flow cytometry (Figure 6F). Both assays revealed that much more NDV was produced *in vivo* in *Jnk2* ^−/−^ mice, as compared with wild-type or *Jnk1* ^−/−^ mice. These data indicate that JNK2 is essential to protect mice against viral invasion.

### JNK2 promotes cell apoptosis, but not innate immune signaling, *in vivo*

**Figure 7:**
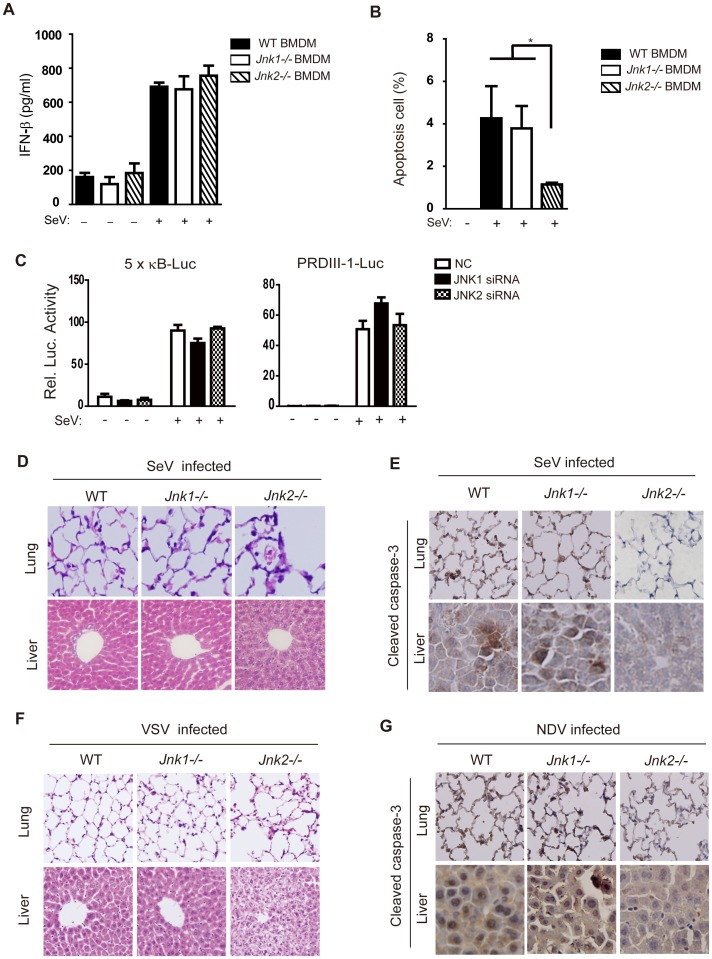
JNK2 protects mice against virus-induced organ injury. (***A***) BMDMs from wild type, *Jnk1^−/−^* or *Jnk2^−/−^* mice were treated with or without SeV (MOI = 1) for 18 hours and then IFN-β production was determined by ELISA. Data are presented as means±SD (n = 3). (***B***) BMDMs from wild type, *Jnk1* ^−/−^ or *Jnk2* ^−/−^ mice were treated with SeV (MOI = 1) for 48 hours, followed by Annexin-V staining analysis. Cells undergoing apoptosis (Annexin-V positive and PI negative) were quantified by flow cytometry. Data are presented as means±SD (n = 3). (***C***) The indicated siRNAs were transfected into HEK293 cells together with NF-κB-Luc reporter or PRDIII-1-Luc reporter plasmids and 24 hours later, cells were infected with or without SeV (MOI = 1). A luciferase assay was performed 12 hours post-infection. Data are presented as means±SD (n = 3). (***D*** and ***E***) Wild type, *Jnk1^−/−^* or *Jnk2^−/−^* mice were intranasally challenged with SeV (10^7^ PFU/g mouse weight). Two days later, lungs and livers were harvested for histochemistry analysis by H&E staining and immunohistochemistry analysis by detecting cleaved caspase-3 staining. (***F*** and ***G***) Mice were infected with VSV or NDV as described in Figure 6. Two days later, lungs and livers were harvested for histochemistry analysis by H&E staining and immunohistochemistry analysis by cleaved caspase-3 staining. Tissue sections were visualized by microscopy (20× objective for lung, 40× objective for liver).

Viral infection induces both innate immunity and cell apoptosis, of which macrophages represent one of the major innate immune cells in early infection. We harvested and cultured bone marrow-derived macrophages (BMDM) from wild type, *Jnk1* ^−/−^ and *Jnk2* ^−/−^ mice. These BMDM were then infected with SeV and their cytokine expression was measured at both the protein and mRNA level (Figure 7A and Figure S6). It was observed that JNK1 and JNK2 only marginally influenced the expression of cytokines induced by innate immune signaling, and there was no difference in IFN-β (Interferon β) protein expression between wild type and JNK2 knockout cells (Figure 7A). Such results suggest that JNK1 and JNK2 do not individually modulate innate immune signaling, but does not rule out the possibility that they could cross-talk with each other.

SeV-induced apoptosis in BMDM was quantitated by Annexin-V/propidium iodide (PI) staining. This apoptosis was markedly attenuated in *Jnk2* ^−/−^ cells compared to both *Jnk1* ^−/−^ cells and wild type cells (Figure 7B). In addition, we confirmed that JNK2 did not directly modulate the IRF3 or NF-κB signaling pathways during virus infection by using the corresponding reporter systems in both JNK1/JNK2 siRNA knock down cells and JNK1/JNK2 overexpressing cells (Figure 7C and Figure S7).

To investigate the *in vivo* function of JNK2 in apoptosis, the lung and liver of *Jnk1* ^−/−^ and *Jnk2* ^−/−^ mice were prepared for immunohistochemistry following SeV, VSV or NDV challenge. It was observed that the lung and liver of *Jnk1* ^−/−^ mice appeared similar to those of the wild type. Remarkably, the tissues of these organs from *Jnk2* ^−/−^ mice displayed severe inflammatory injury, particularly around the blood vessels in the liver (Figure 7D and 7F). Morphologically, those cells appeared necrotic, based on the absence of staining to detect caspase-3 activation in *Jnk2* ^−/−^ samples, which was evident in the wild type and *Jnk1* ^−/−^ samples (Figure 7E and 7G). Overall, this data clearly demonstrates that JNK2 plays an indispensable role in the initiation of virus-induced apoptosis, without which cells undergo necrosis and trigger inflammatory damages.

## Discussion

Viral infection induces innate immunity and apoptosis, which represent effective means for the host to restrict the spread of microbial pathogens. The essential function of MAVS has been well documented in mediating RIG-I/MDA5 innate immune signaling. In this work, we demonstrate that MAVS is also critical for cell apoptosis upon viral infection and highlight the dual function of MAVS in innate immunity and apoptosis.

**Figure 8:**
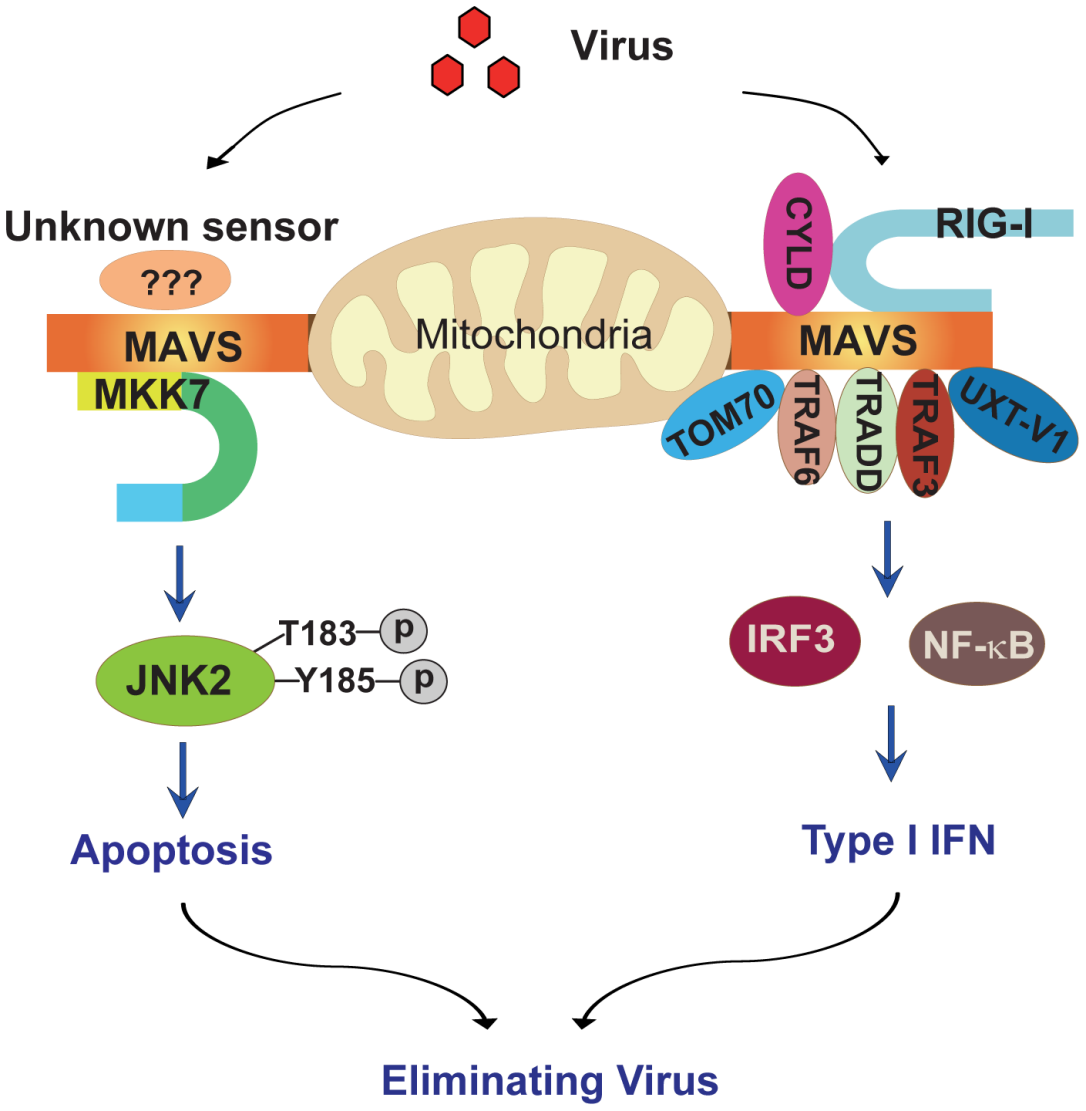
Schematic diagram of the MAVS-MKK7-JNK2 apoptosis signaling pathway. MAVS is a critical converging point of the host response to RNA virus infection. By engaging RIG-I or MDA5, MAVS forms a mitochondrial signalosome, dynamically composed of TRAF2/3/6, TRADD, TOM70, UXT-V1 and so on. This signalosome leads to the activation of TBK1 and IKK and then the ultimate induction of type 1 interferon and inflammatory cytokines, thereby establishing the antiviral state. In addition, MAVS also recruits MAPK kinase MKK7 onto the mitochondria via protein-protein interaction. MKK7 subsequently induces the phosphorylation of JNK2 at its threonine-183 and tyrosine-185 positions. Finally, JNK2 initiates cell apoptosis to sacrifice the virus-infected host cell, thereby dampening virus-induced inflammatory injury.

Our current study defines a novel apoptotic signaling pathway (Figure 8): upon viral infection, MAVS recruits the MAPK kinase MKK7 onto mitochondria, MKK7 phosphorylates and activates JNK2, and JNK2 then initiates the corresponding cell apoptosis. Apoptosis is essential for sacrificing virus-infected cells and dampening the detrimental inflammation, as evidenced by the response of *Jnk2* ^−/−^ mice to viral infection. This study highlights the convergence of innate immunity and apoptosis on MAVS during viral infection, further substantiating the notion that the mitochondrial outer membrane is the critical signaling platform for cellular stress responses.

C-Jun N-terminal kinases (JNKs) play important roles in death receptor-initiated extrinsic as well as mitochondria-initiated intrinsic apoptotic pathways, in response to stress stimuli, such as cytokines, DNA damages, heat shock and osmotic stress *et.al*. They are also known to modulate cell proliferation and differentiation [19], [20]. JNKs can quickly induce the expression of pro-apoptotic genes via their activation of specific transcription factors. In addition, they can directly regulate the pro- and/or anti-apoptotic activities of mitochondria-related proteins via distinct post-translational phosphorylation [20]. Whether the activation of JNKs leads to cell proliferation or apoptosis is dependent on the stimuli and cell type involved [21]. Further investigation is needed to dissect the manner by which JNK2 triggers the relevant apoptosis effector mechanism, downstream of MAVS-mediated signaling. One possibility is that JNK2 is integrated into the well-characterized effector responses, such as the induction of pro-apoptotic genes, cleavage of Bid, BAD/Bim phosphorylation, or the Bax clustering on mitochondria *et.al*. It is also intriguing to probe the potential cross-talk among these various apoptotic pathways.

JNK1 and JNK2 are expressed in all cells and tissues, whereas JNK3 expression is predominantly localized in the brain [22]. The functions of JNK1 and JNK2 are largely redundant in canonical apoptosis signaling, with JNK1 as a major player [23], [24]. Using both *in vitro* and *in vivo* approaches, we clearly differentiate the role of JNK1 and JNK2 in virus-induced apoptosis, establishing the indispensable role of JNK2 in the MAVS-mediated apoptosis. In addition, we rule out the potential role of p38 and ERK in this apoptosis.

It remains to be addressed why MAVS selectively interacts with MKK7 instead of MKK4. Likewise, it is unknown why JNK2 was chosen for this pathway instead of JNK1. We speculate that there might be yet-to-discovered scaffold proteins responsible for the selectivity. For example, JNK-interacting protein 1 (JIP1) has been shown to enhance the activation of JNKs via MLK3 and MKK7, whereas JIP2 specifically interacts with MKK7 and not MKK4. JNK3 has been shown to have more affinity for JSAP1/JIP3 than JNK1 or JNK2 [25].

During our preparation of this manuscript, MAVS was reported to be activated by La Crosse virus (LACV) infection and to upregulate the adaptor protein SARM1, which is related with neuronal death [17]. Although the authors demonstrated that MAVS was associated with SARM1-mediated cell death in neurons, no specific molecular mechanism was presented in their study. Given the tissue-specific expression of JNK3 in neurons, it is reasonable to presume that JNK3 probably plays some role in MAVS-SARM1 mediated neuronal death. We believe that the MAVS-MKK7-JNK may represent a general mechanism for the host to quickly respond to viral infections. It is also unexpected that members of the JNK family display such delicate and clear-cut differences in terms of their biological functions.

With the exception of MAVS, there are no overlapping signaling proteins between the innate and apoptosis signaling pathways during RNA virus infection. The loss of JNK1/2 did not affect the RNA-virus-induced IRF3 or NF-κB activation. Unexpectedly, RIG-I/MDA5 was dispensable for the virus-induced apoptosis, suggesting the existence of other sensors upstream of MAVS. This is analogous to the cellular recognition of DNA viruses. Several DNA sensors have been reported recently, including Absent In Melanoma 2 (AIM2) [26], DNA-dependent activator of interferon regulatory factors (DAI) [27], DEAD box polypeptide 41 (DDX41) [28], interferon inducible protein 16 (IFI16) [29], and cGAMP synthase (cGAS) [30], [31]. Each of these sensors activate the endoplasmic reticulum protein called stimulator of interferon genes (STING). Like MAVS, STING activates both IKK and IRF3, thereby turning on the NF-κB and IRF3 signaling pathways [32], [33]. In the current study, we established the role of MAVS-MKK7-JNK2 signaling in mediating RNA virus-induced apoptosis. Future studies will address whether STING plays a role in JNK activation during DNA virus-induced apoptosis. In addition, it remains intriguing to examine potential sensor(s) for RNA virus-induced apoptosis.

## Materials and Methods

### Ethics statement

C57BL/6 mice were purchased from the Shanghai SLAC Laboratory Animal Company. The mice were maintained under specific pathogen-free (SPF) conditions at the Shanghai Institute of Biochemistry and Cell Biology. All animals used in this study were 4∼8 weeks of age. Animal experiments were carried out in strict accordance with the regulations in the Guide for the Care and Use of Laboratory Animals issued by the Ministry of Science and Technology of the People's Republic of China. The protocol was approved by the Institutional Animal Care and Use Committee of the Shanghai Institute of Biochemistry and Cell Biology, Chinese Academy of Sciences (Permit Number: IBCB0027 Rev).

### Virus infection of mice

*Jnk1* ^−/−^ and *Jnk2* ^−/−^ mice [34], [35] were kindly provided by Dr. Lijian Hui. These stains were maintained on a C57BL/6 background. For infection, 4∼8 weeks old mice were narcotized and then intranasally inoculated with SeV, VSV or NDV-GFP (volume≤20 µl). Negative controls included water and PBS. Clinical symptoms were observed 2∼7 days post inoculation, and the following analyses were performed.

### Histology

After sacrifice, mice were perfused sufficiently with PBS, and then lung and liver tissues were fixed in 4% paraformaldehyde (in PBS) for 12 hours. Tissue sections were prepared at the Core Facility for Cell biology, Shanghai Institute of Biochemistry and Cell Biology and then hematoxylin and eosin (H&E) staining was performed. For immunohistochemistry of cleaved caspase-3, the EnVision FLEX Systems Kit (Dako) was used.

### Fluorescence stereomicroscopy

Two days post NDV-GFP infection (10^7^ PFU/mouse), mice were sacrificed and the lungs perfused with PBS. *In situ* NDV-GFP was observed using Olympus SZX16 fluorescence stereomicroscopy.

### Viral titer assay

After VSV or NDV-GFP infection for the indicated days, peripheral blood was collected to obtain serum. For VSV titer assay, sera was added into culture media of HEK293 cells and the viral titer was determined by a standard plaque assay [8]. For NDV-GFP title assay, equal amounts of sera were added into culture media of HEK293 cells and cultured for 24 hours. GFP positive cells (NDV-GFP infected cells) were analysis by both fluorescent microscopy and flow cytometry.

### Cell culture

*Mavs* ^−/−^ MEFs were provided by Dr. Zhengfan Jiang. *Mkk4/7* ^−/−^ and *Mkk3/6* ^−/−^ MEFs were provided by Dr. Jiahuai Han and *Rig-i* ^−/−^ MEF cells were provided by Dr. Deyin Guo and Dr. Hong-Bing Shu. HEK293, HEK293T and L929 cell lines were obtained from the American Type Culture Collection (ATCC). For bone marrow-derived macrophages (BMDM), femurs were aseptically harvested from mice. Bone marrow cells were flushed from the bones and then cultured in RPMI 1640 media (20% FBS, 2 mM L-glutamine, 50 mM 2-mercaptoethanol) with 40 ng/ml macrophage colony stimulating factor (M-CSF). The medium was refreshed every two days and the cells differentiated to BMDMs in one week. All cells were maintained in a humidified 5% CO_2_ incubator at 37°C.

### Antibodies and reagents

Recombinant Human TNF-α was purchased from R&D Systems. Cycloheximide (CHX) was purchased from Sigma. The following antibodies were used for western blot or immunoprecipitation: anti-β-actin (A5316, Sigma), normal mouse IgG (sc-2025, Santa Cruz Biotechnology), normal rabbit IgG (sc-2027, Santa Cruz Biotechnology), anti-HA (sc-7392, Santa Cruz Biotechnology), anti-Flag (F1804, Sigma), anti-Tom20 (11802-1-AP, Proteintech; sc-17764, Santa Cruz Biotechnology), anti-Caspase-3 (9662, Cell Signaling; 9661, Cell Signaling), anti-PARP (sc-7150, Santa Cruz Biotechnology; 9542, Cell Signaling), anti-MAVS (generated by this laboratory and also purchased from Cell Signaling–3993), anti-JNK (sc-571, Santa Cruz Biotechnology), anti-p-JNK (9255, Cell Signaling), anti-p38 (sc-7149, Santa Cruz Biotechnology), anti-p-p38 (9211, Cell Signaling), anti-ERK (9102, Cell Signaling), anti-p-ERK (9101, Cell Signaling), anti-TBK1 (sc-73115, Santa Cruz Biotechnology), anti-ISG15 (M24004, Abmart), anti-ISG60 (15201-1-AP, Proteintech), anti-MKK4 (sc964, Santa Cruz Biotechnology), anti-MKK7 (generated by this laboratory and also purchased from Abcam–ab52618), anti-p-IRF3 (4947, Cell Signaling), anti-TRAF2 (sc-876, Santa Cruz Biotechnology), anti-TRAF3 (sc-1828, Santa Cruz Biotechnology), anti-TRADD (sc-46653, Santa Cruz Biotechnology), anti-RIG-I (AB54008, Shanghai Sangon Biotech; 4520, Cell Signaling), anti-MDA5 (5321, Cell Signaling), anti-MKK3 (5674, Cell Signaling) and anti-MKK6 (9264, Cell Signaling). Mitotracker Red was obtained from Molecular Probes and DAPI was obtained from Life Technologies. Annexin-V-FLUOS Staining Kit was purchased from Roche (11858777001). Sendai virus (SeV), Vesicular stomatitis virus (VSV) and Newcastle disease virus-GFP (NDV-GFP) were kindly provided by Drs. Hong-Bing Shu (Wuhan University) and Zhigao Bu (Chinese Academy of Agricultural Sciences).

### Plasmids, siRNA oligos, and cell transfection

Human JNK1, JNK2, RIG-I, MKK4, MKK7, IKKα, IKKε, TBK1 and MAVS cDNAs were cloned from a human thymus plasmid cDNA library (Clontech) using standard PCR techniques and then sub-cloned into the indicated vectors. Mutants were generated by using a Quickchange XL (Stratagene). MAVS-ΔTM was truncated of its C-terminal transmembrane domain (514–540 aa). MKK7-Δ3D was truncated of its N-terminal docking domain (1–85 aa), MKK7-ΔDVD was truncated of its domain of versatile docking (378–400 aa), and MKK7-PK only maintained its protein kinase domain (80–380 aa). The siRNA oligos were synthesized by GenePharma:

JNK1 siRNA: 5′GCCCAGUAAUAUAGUAGUATT3′, 5′GAGCUAGUUCUUAUGAAAUTT3′;

JNK2 siRNA: 5′GUUGCAGUCAAGAAACUAATT3′, 5′GUGAACUUGUCCUCUUAAATT3′;

MKK4 siRNA: 5′ AAUGCGGAGUAGUGAUUGTT3′, 5′GAUUUCACUGCAGAGGACUUTT3′;

MKK7 siRNA: 5′ UAAGCUACUUGAACACAGCTT3′, 5′GAACAAGGAGGAGAACAATT3′;

RIG-I siRNA: 5′ ACGGAUUAGCGACAAAUUUAATT3′, 5′GAAUUUAAAACCAGAAUUAUCTT3′;

MDA5 siRNA: 5′AUCACGGAUUAGCGACAAATT3′, 5′GAAUAACCCAUCACUAAUATT3′

TRAF2 siRNA: 5′ CGACAUGAACAUCGCAAGCTT3′, 5′AGGAGCAUUGGCCUCAAGGATT3′;

TRAF3 siRNA: 5′ GGAGGUUACAAGGAAAAGUTT 3′, 5′ GAAGGUUUCCUUGUUGCAGAAUGAA3′;

TRADD siRNA: 5′ GGAGGAUGCGCUGCGAAAUUU3′, 5′ AACUGGCUGAGCUGGAGGAUGTT 3′;

MAVS siRNA: 5′CCACCUUGAUGCCUGUGAATT3′, 5′CAGAGGAGAAUGAGUAUAATT3′;

Negative control siRNA, 5′UUCUCCGAACGUGUCACGUTT3′.

Cells were transfected with siRNA oligos using Lipofectamine 2000 and then incubated for 48 hours before further analysis. The plasmids were introduced into cells using Lipofectamine 2000, and then cells were cultured for 24 hours before further analysis.

### Real-time PCR

Total cellular RNA was isolated using Trizol (Invitrogen) according to the manufacturer's instructions. Reverse transcription of purified RNA was performed using oligo(dT) primers. The quantification of gene transcripts was performed by real-time PCR using SYBR Green PCR mix (Applied Biosystems). All values were normalized to the level of β-actin mRNA. The primers used are listed below:

β-actin, sense (AAAGACCTGTACGCCAACAC) and antisense (GTCAT ACTCCTGCTTGCTGAT);

IFN-β, sense (AGATCAACCTCACCTACAGG) and antisense (TCAGAAACACTGTCTGCTGG);

ISG15, sense (GGAACGAAAGGGGCCACAGCA) and antisense (CCTCCATGGGCCTTCCCTCGA);

ISG56, sense (AGTGCA GGCAGAAATTCACC) and antisense (AGCAGTCAGTAGTTTCCTCC);

IL-6, sense (GAGAGGAGA CTTCACAGAGG) and antisense (GTACTCCAGAAGACCAGAGG);

IL-12, sense (GCTTCTTCATCAGGGACATC) and antisense (GTCAGGGAGAAGTAGGAATG);

### Annexin-V staining and flow cytometry

Both floating and adherent cells were collected at the designated time points and stained with Annexin-V (Roche, 11858777001) according to the manufacturer's instructions. Cell apoptosis was detected using a FACS Calibur (BD Biosciences) and the data was analyzed using Flowjo software (Tree Star).

### Western blot and immunoprecipitation

Cell pellets were collected and resuspended in RIPA buffer (50 mM Tris-HCl, pH 7.4, 150 mM NaCl, 1 mM EDTA, 0.5% NP40, 0.25% Na-deoxycholate, 1 mM Na_3_VO4, 0.1 mM PMSF, Roche complete protease inhibitor set) for immunoprecipitation, or in RIPA buffer plus 0.1% SDS for western blot analysis. The resuspended cell pellet was vortexed for 20 seconds and then incubated on ice for 20 minutes, followed by centrifugation at 20,000 g for 20 minutes. Supernatants were collected for subsequent immunoprecipitation or western blot analysis

For immunoprecipitation, cell lysates were pre-cleared with Protein A/G Plus-Agarose (Santa Cruz Biotech) at 4°C for 2 hours, then antibody or control IgG was added and incubated overnight. The next day, cell lysates were incubated for an additional 2 hours since the Protein A/G Plus-Agarose beads were added. The beads were washed with TBS buffer containing 0.5% NP40, boiled using 1× SDS loading buffer, and the supernatants were loaded for western blot analysis.

### Subcellular fractionation

HEK293 or MEF cells were washed with cold PBS and lysed using a Dounce Homogenizer in homogenization buffer (210 mM sucrose, 70 mM mannitol, 1 mM EDTA, 1 mM EGTA, 1.5 mM MgCl_2_, 10 mM HEPES [pH 7.2]). The homogenate was centrifuged at 500 g for 10 minutes, and the pellet was discarded as crude nuclei. The supernatant was centrifuged at 5,000 g for 10 minutes to precipitate crude mitochondria; the supernatants were collected as the cytosolic fraction and the precipitate was lysed by RIPA buffer to obtain the mitochondrial fraction.

### Confocal imaging

MEF cells were plated on coverslips in 12-well plates and transfected with the indicated plasmids. Twenty-four hours later, cells were treated with or without SeV (MOI = 1) for 6 hours. To label mitochondria in a specific experiment, cells were incubated with 250 nM Mitotracker Red for 30 min at 37°C. Coverslips with the cells were washed once with PBS and fixed in 3.7% formaldehyde in PBS for 15 minutes. After permeabilization with Triton X-100 (0.25%) in PBS for 15 minutes, cells were blocked with PBS containing BSA (5%) for 1 hour and then incubated with primary antibodies for 1 hour. After three separate washes, cells were incubated with secondary antibody for another hour and then stained with DAPI for 2 minutes. The coverslips were washed extensively and fixed on slides. Images were captured using a Leica laser scanning confocal microscope (Leica TCS SP2 AOBS).

### Measurement of IFN-β protein

BMDMs were isolated from wild-type, *Jnk1−/−* and *Jnk2−/−* mice and cultured as described previously. Cell culture supernatants were collected at 18 hours post virus infection and were analyzed for IFN-β production by ELISA (PBL Biomedical Laboratories).

### Luciferase assay

Luciferase reporter assays were performed as described previously [36].

### Statistics

All experiments in this study were performed independently at least three times. For western blots, fluorescence images and histology sections, one representative result has been shown. For real-time PCR, ELISA, Luciferase, Annexin-V and viral titer assays, the data are represented by three independent experiments. The scale of animal experiments has been instructed in figure legends. Student's t-test was used for the comparison of three independent treatments. For all tests, a p value <0.05 was considered statistically significant.

### Accession numbers

The GenBank (http://www.ncbi.nlm.nih.gov/Genbank) accession numbers for the genes and gene products discussed in this paper are: RIG-I (NM_014314.3→NP_055129.2; NM_172689.3→NP_766277.3), MDA5 (NM_022168.3→NP_071451.2), MAVS (NM_020746.4→NP_065797.2; NM_001206385.1→NP_001193314.1), TBK1 (NM_013254.3→NP_037386.1; NM_019786.4→NP_062760.3), IKKα (NM_001278.3→NP_001269.3), IKKε (NM_014002.3→NP_054721.1), JNK1 (NM_139046.2→NP_620634.1; NM_016700.4→NP_057909.1), JNK2 (NM_002752.4→NP_002743.3; NM_207692.2→NP_997575.2), MKK3 (NM_008928.4→NP_032954.1), MKK4 (NM_003010.3→NP_003001.1; NM_009157.4→NP_033183.1), MKK6 (NM_011943.2→NP_036073.1), MKK7 (NM_145185.2→NP_660186.1; NM_001042557.2→NP_001036022.1).

## Supporting Information

Figure S1 (***A***) Negative control, RIG-I or MDA5 siRNAs were introduced into HEK293 cells. Fourty-eight hours later, cells were treated with SeV (MOI = 1) for the indicated times. Phosphorylated JNK was determined by western blot analysis. RIG-I and MDA5 were also probed for siRNA silencing efficiency. (***B***) Wild type or *Rig-i^−/−^* MEF cells were stimulated with SeV (MOI = 4) for the indicated times. Cleaved PARP and cleaved caspase-3 were determined by western blot analysis.
(TIF)

Figure S2 (***A*** and ***B***) HEK293 cells were treated with SeV (MOI = 1) with or without the caspase inhibitor Z-VAD (10 µM). Cell lysates were collected for western blot analysis for PARP, cleaved caspase-3, MAVS, p-JNK and β-actin to measure cell apoptosis. (***C***) Wild type, *Jnk1* ^−/−^ or *Jnk2* ^−/−^ MEF cells were treated with VSV (MOI = 1) for the indicated times and the cell lysates were collected for western blot analysis.
(TIF)

Figure S3 TRAF2, TRAF3,TRADD or control siRNAs were introduced into HEK293 cells for 48 hours, and then cells were treated with SeV (MOI = 1) for the indicated times. Cell lysates were collected for western blot analysis to measure phosphorylated JNK, cleaved PARP, TRAF2, TRAF3 and TRADD.
(TIF)

Figure S4 HEK293T cells were transfected with the indicated combination of plasmids for 24 hours. Cell lysates were immunoprecipitated with anti-HA antibody, followed by western blot with anti-HA and anti-Flag antibodies, respectively.
(TIF)

Figure S5 Diagram for MKK7 truncation mutants. 3D domain, 3 docking domains; DVD domain, domain for versatile docking; PK domain, protein kinase domain.
(TIF)

Figure S6 (***A***) BMDMs from wild type and Jnk1^−/−^ mice were treated with SeV (MOI = 1) for 12 hours. The induction of the indicated mRNAs were measured by real-time PCR. Data are presented as means±SD (n = 3). (***B***) BMDMs from wild type and Jnk2^−/−^ mice were treated with SeV (MOI = 1) for 12 hours. The induction of the indicated mRNAs were measured by real-time PCR. Data are presented as means±SD (n = 3). IL-6, Interleukin 6. IL-12, Interleukin 12.
(TIF)

Figure S7 (***A***) HEK293 cells were transfected with the indicated plasmids with PRDIII-1-luc reporters and then treated with or without SeV (MOI = 1) for 12 hours. A luciferase assay was then performed. Data are presented as means±SD (n = 3). (***B***) HEK293 cells were transfected with the indicated plasmids with 5xκB-luc reporters and then treated with or without SeV (MOI = 1) for 12 hours. A luciferase assay was then performed. Data are presented as means±SD (n = 3).
(TIF)
