# Supplementary figures and images for: MAVS-MKK7-JNK2 Defines a Novel Apoptotic Signaling Pathway during Viral Infection
Source: PLoS Pathog. 2014 Mar 20;10(3):e1004020. doi: 10.1371/journal.ppat.1004020 (PMC3961361; doi:10.1371/journal.ppat.1004020)

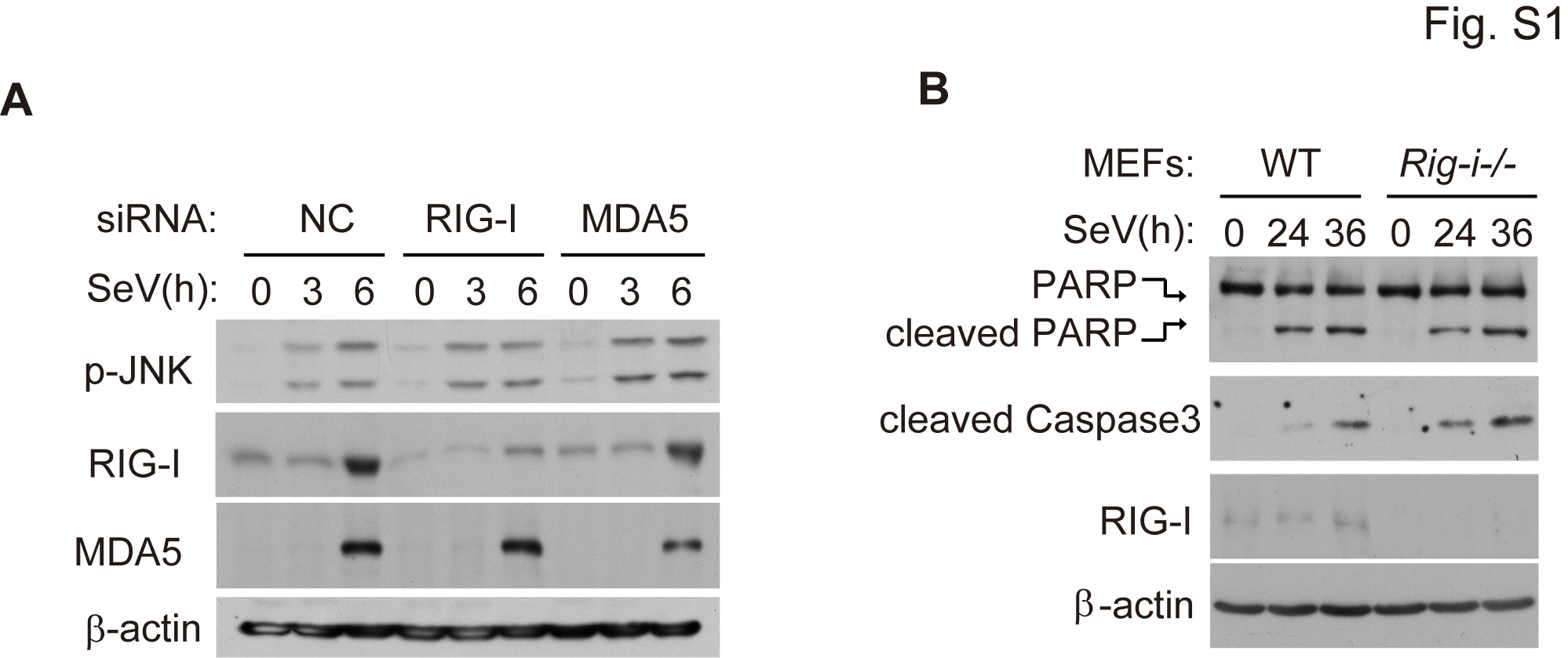

Supplement: Figure S1 — (A) Negative control, RIG-I or MDA5 siRNAs were introduced into HEK293 cells. Fourty-eight hours later, cells were treated with SeV (MOI = 1) for the indicated times. Phosphorylated JNK was determined by western blot analysis. RIG-I and MDA5 were also probed for siRNA silencing efficiency. (B) Wild type or Rig-i−/− MEF cells were stimulated with SeV (MOI = 4) for the indicated times. Cleaved PARP and cleaved caspase-3 were determined by western blot analysis. (TIF) [file ppat.1004020.s001.tif]

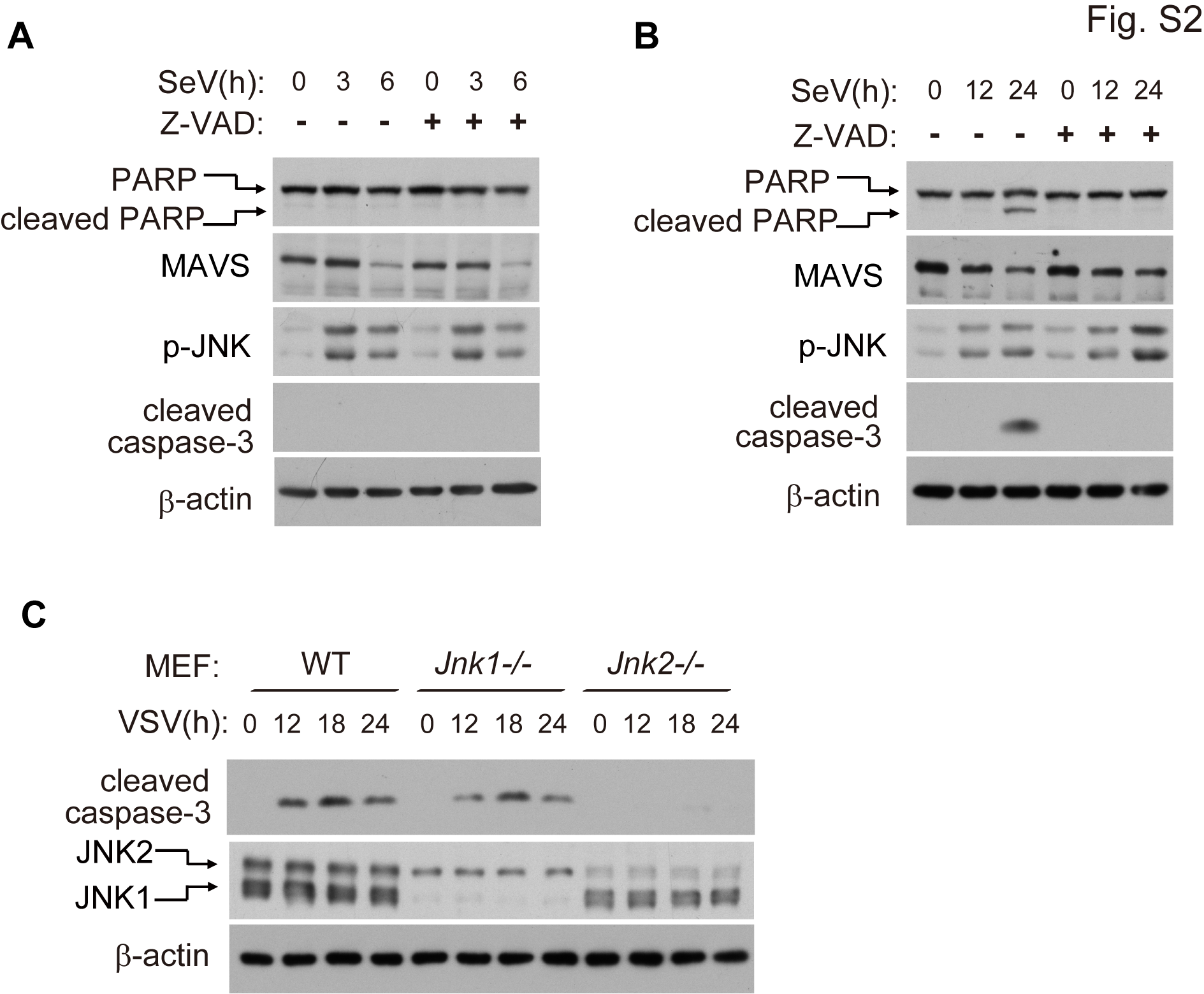

Supplement: Figure S2 — (A and B) HEK293 cells were treated with SeV (MOI = 1) with or without the caspase inhibitor Z-VAD (10 µM). Cell lysates were collected for western blot analysis for PARP, cleaved caspase-3, MAVS, p-JNK and β-actin to measure cell apoptosis. (C) Wild type, Jnk1 −/− or Jnk2 −/− MEF cells were treated with VSV (MOI = 1) for the indicated times and the cell lysates were collected for western blot analysis. (TIF) [file ppat.1004020.s002.tif]

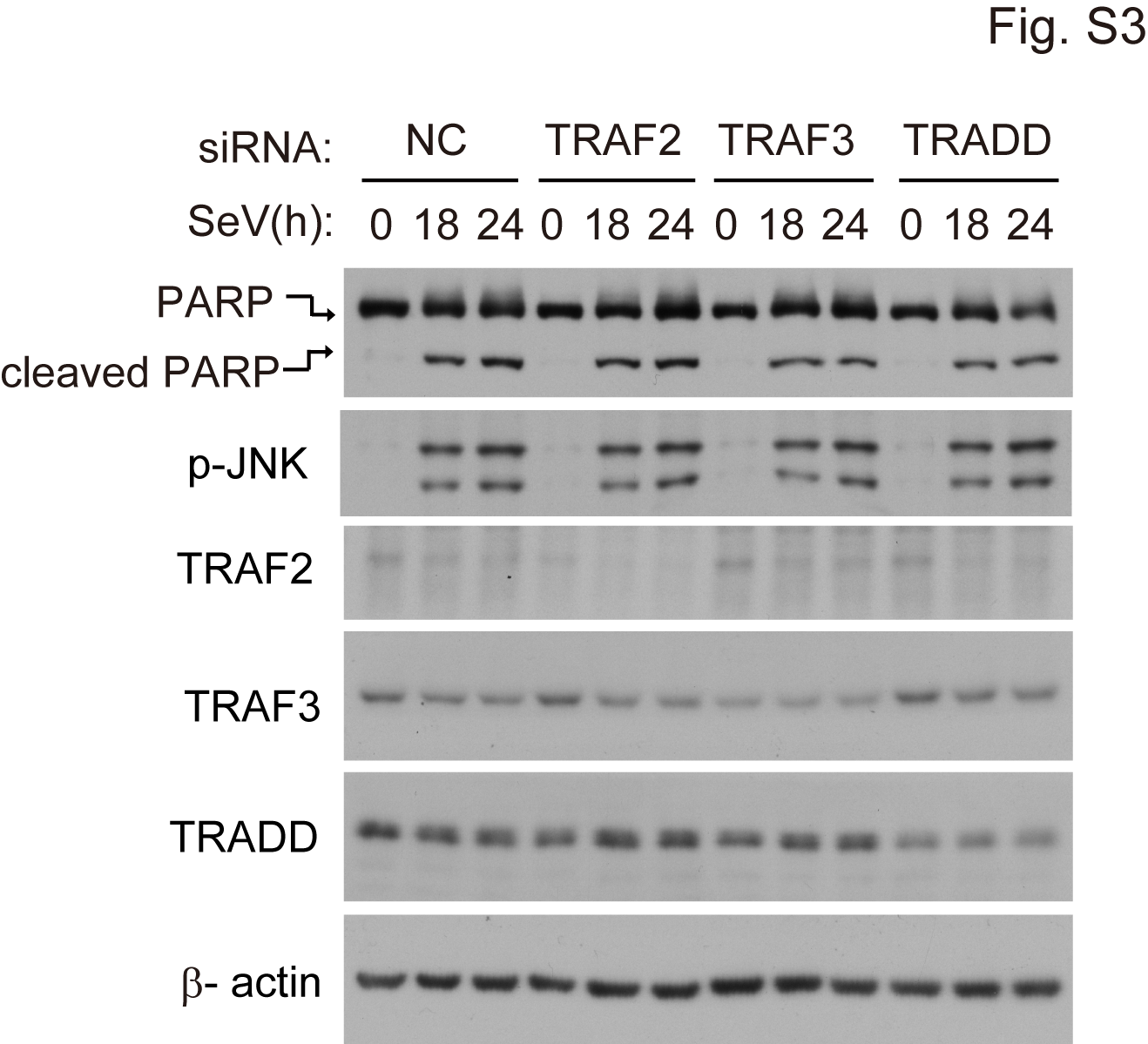

Supplement: Figure S3 — TRAF2, TRAF3,TRADD or control siRNAs were introduced into HEK293 cells for 48 hours, and then cells were treated with SeV (MOI = 1) for the indicated times. Cell lysates were collected for western blot analysis to measure phosphorylated JNK, cleaved PARP, TRAF2, TRAF3 and TRADD. (TIF) [file ppat.1004020.s003.tif]

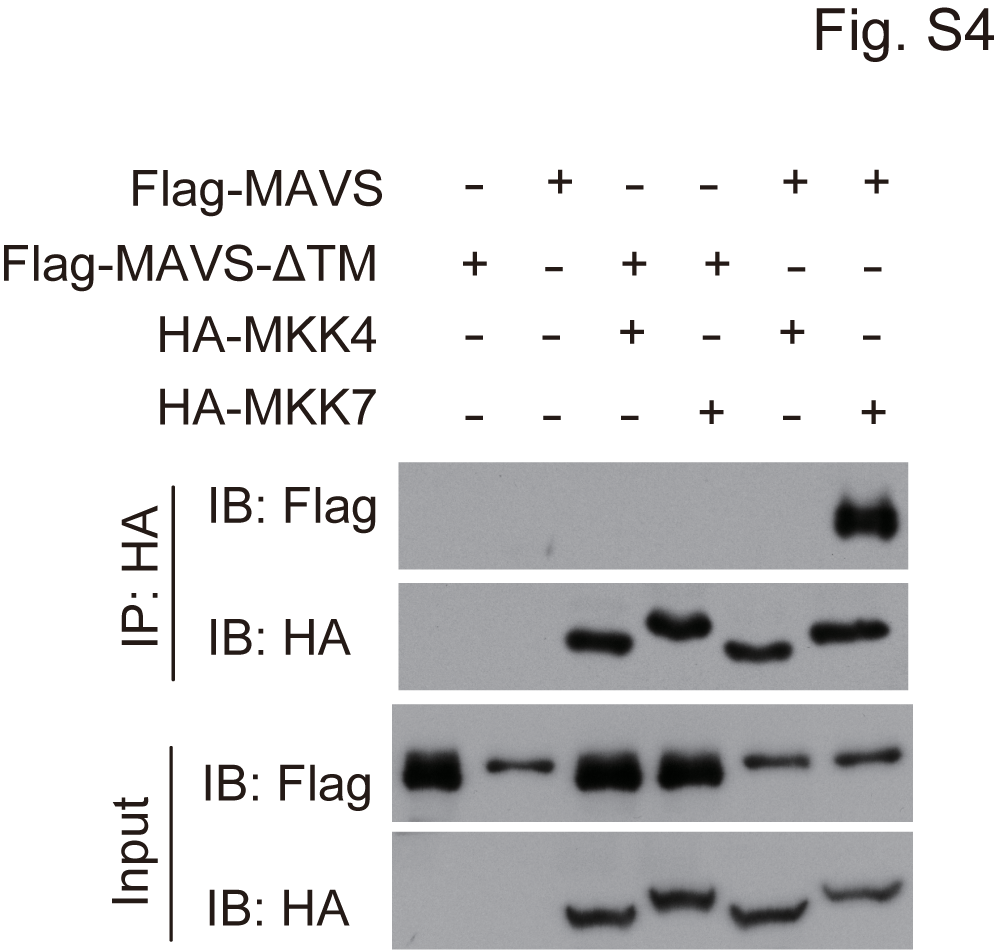

Supplement: Figure S4 — HEK293T cells were transfected with the indicated combination of plasmids for 24 hours. Cell lysates were immunoprecipitated with anti-HA antibody, followed by western blot with anti-HA and anti-Flag antibodies, respectively. (TIF) [file ppat.1004020.s004.tif]

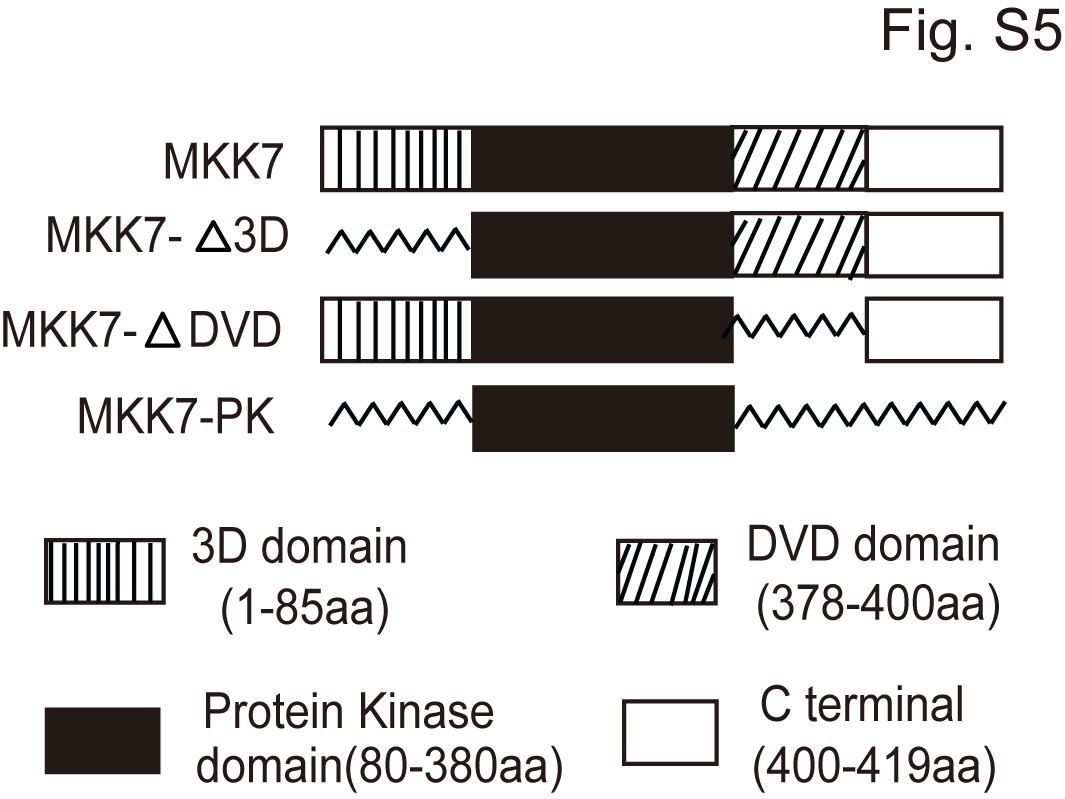

Supplement: Figure S5 — Diagram for MKK7 truncation mutants. 3D domain, 3 docking domains; DVD domain, domain for versatile docking; PK domain, protein kinase domain. (TIF) [file ppat.1004020.s005.tif]

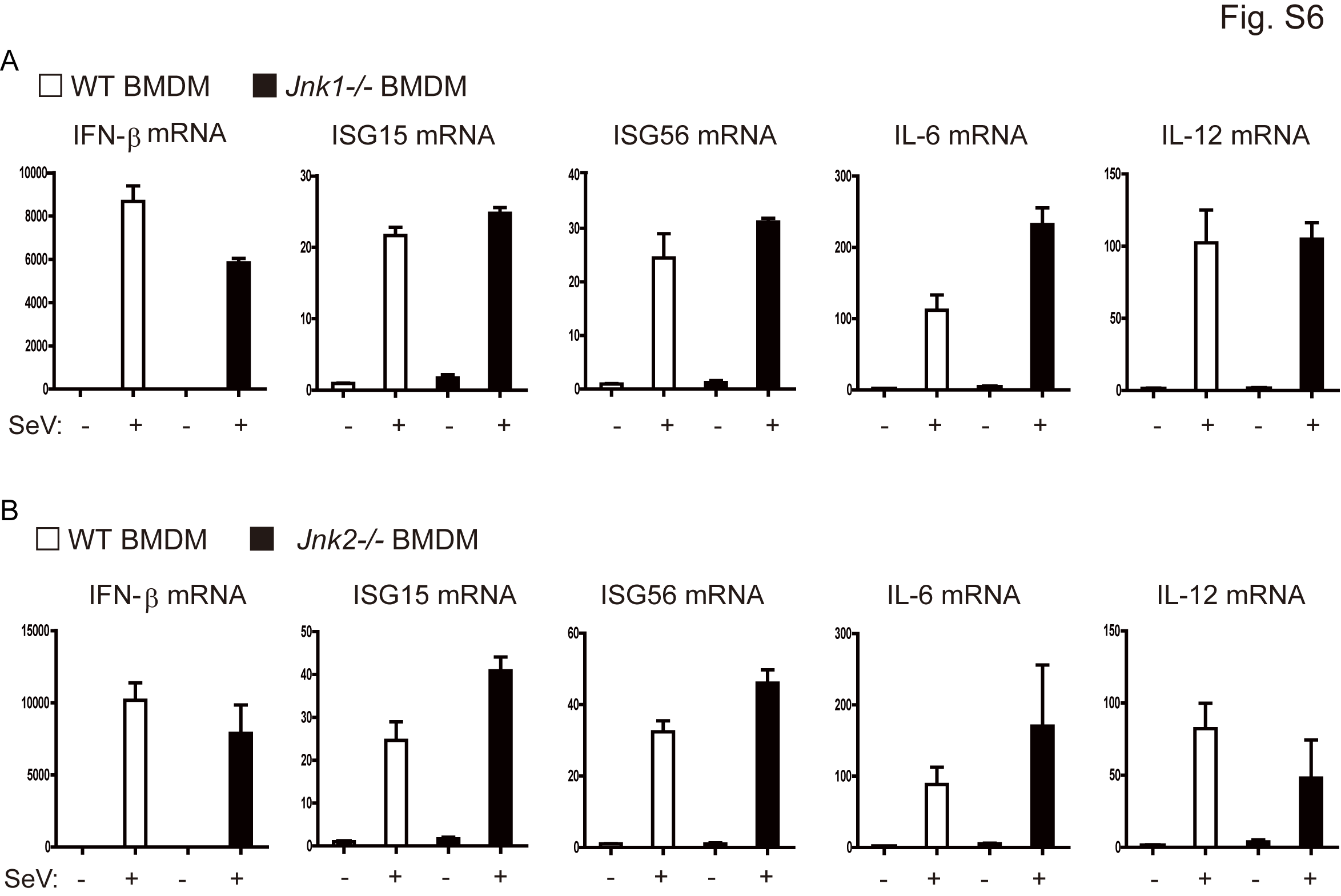

Supplement: Figure S6 — (A) BMDMs from wild type and Jnk1−/− mice were treated with SeV (MOI = 1) for 12 hours. The induction of the indicated mRNAs were measured by real-time PCR. Data are presented as means±SD (n = 3). (B) BMDMs from wild type and Jnk2−/− mice were treated with SeV (MOI = 1) for 12 hours. The induction of the indicated mRNAs were measured by real-time PCR. Data are presented as means±SD (n = 3). IL-6, Interleukin 6. IL-12, Interleukin 12. (TIF) [file ppat.1004020.s006.tif]

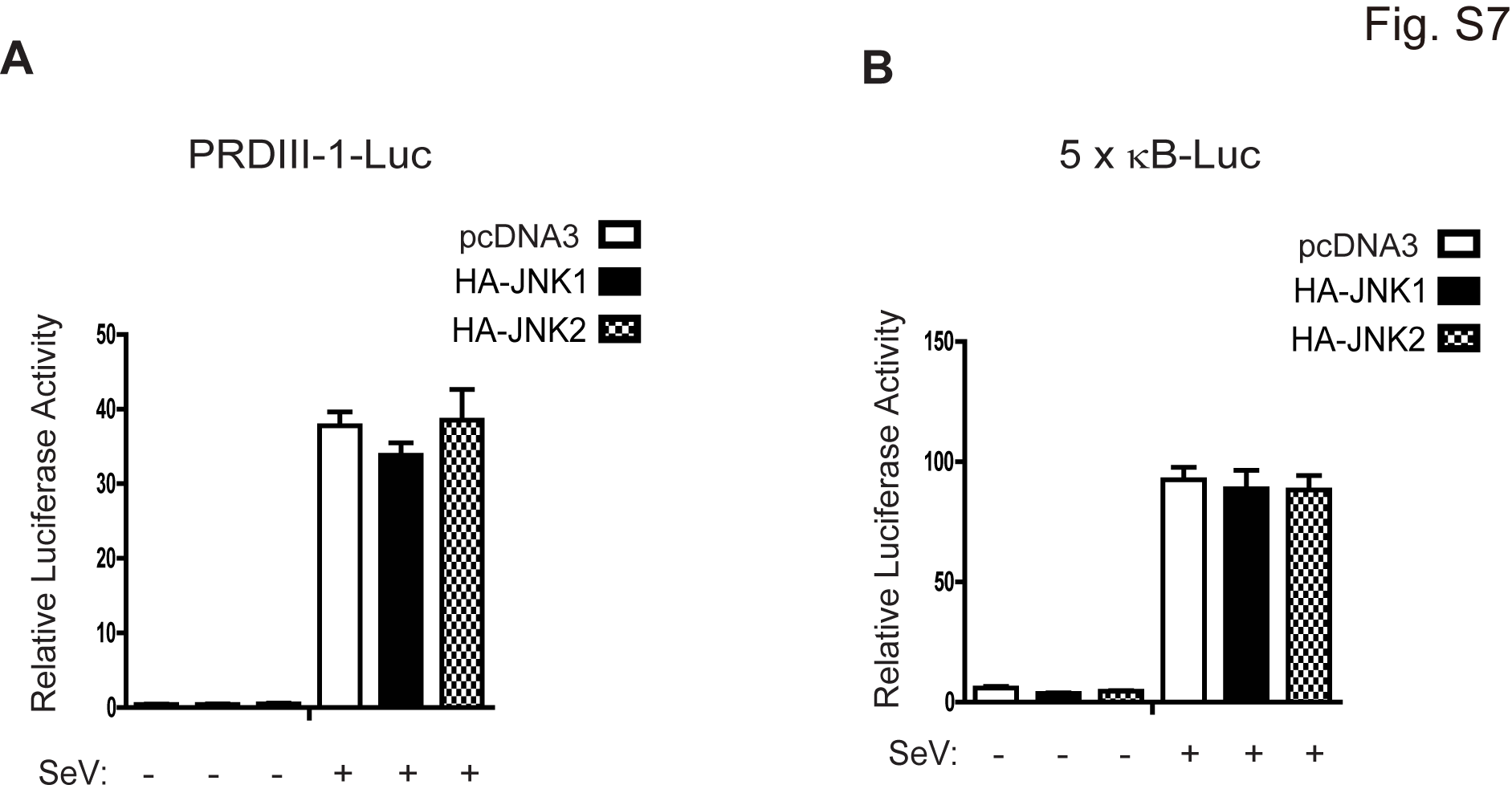

Supplement: Figure S7 — (A) HEK293 cells were transfected with the indicated plasmids with PRDIII-1-luc reporters and then treated with or without SeV (MOI = 1) for 12 hours. A luciferase assay was then performed. Data are presented as means±SD (n = 3). (B) HEK293 cells were transfected with the indicated plasmids with 5xκB-luc reporters and then treated with or without SeV (MOI = 1) for 12 hours. A luciferase assay was then performed. Data are presented as means±SD (n = 3). (TIF) [file ppat.1004020.s007.tif]
